# Improving Antibiotic Stewardship among Asymptomatic Newborns Using the Early-onset Sepsis Risk Calculator

**DOI:** 10.1097/pq9.0000000000000459

**Published:** 2021-08-26

**Authors:** Marty Ellington, Kavita Kasat, Kim Williams, Victoria Reichman, Guillaume Stoffels, Tung Ming Leung, Ana Degoy, Rebecca Fisk, Luisa Gonzalez-Ballesteros, Dinabel Peralta-Reich, Aaron Potash, Kavya Rao

**Affiliations:** From the * Lenox Hill Hospital, New York, N.Y.; † Zucker School of Medicine at Hofstra/Northwell

## Abstract

**Introduction:**

Neonatologists have long struggled with identifying and treating early-onset sepsis (EOS) without overexposing newborns to unnecessary antibiotics.

**Methods:**

In January 2016, we instituted an EOS protocol based mainly on the 2012 AAP guidelines. We subsequently conducted 2 additional plan-do-study-act cycles to decrease antibiotic usage by integrating the EOS risk calculator into our algorithm. For the periods January 2016–June 2017 (period 1), June 2017–February 2018 (period 2), and February 2018–December 2018 (period 3), we tracked all asymptomatic newborns older than 36 weeks, including those admitted to the neonatal intensive care unit for evaluation of EOS. We monitored the monthly variation in asymptomatic newborns older than 36 weeks who received antibiotics using statistical process control. The number of asymptomatic infants treated with antibiotics during the 3 periods was analyzed. Pairwise comparisons were made using post hoc chi-square analysis.

**Results:**

The addition of the EOS calculator score to our guidelines reduced the number of asymptomatic infants older than 36 weeks treated with antibiotics by 73% (*P* < 0.0001). Adopting the EOS calculator score after clinical examination further reduced the number of infants treated by 89% (*P* < 0.0001). For period 1, the percentage of asymptomatic infants older than 36 weeks treated with antibiotics was 4.3%; for period 2, it was 1.16%, and for period 3, it was 0.12% (*P* < 0.0001).

**Conclusions:**

The addition of the EOS calculator score to our AAP-based guidelines reduced antibiotic use among asymptomatic infants older than 36 weeks by 73%. Further adoption of the EOS calculator score after the clinical examination enabled our team to defer antibiotics in almost all asymptomatic infants safely.

## INTRODUCTION

Like many other newborn delivery services, the Lenox Hill Hospital Newborn Service has wrestled with the clinical dilemma of managing asymptomatic term and near-term newborns at risk for neonatal early-onset sepsis (EOS). Lenox Hill Hospital is a tertiary hospital with a 28-bed neonatal intensive care unit (NICU) and approximately 4,000 newborn deliveries annually. Historically, asymptomatic term and near-term infants with sepsis risk factors (maternal fever or suspected chorioamnionitis, or prolonged rupture of membranes) were admitted to the NICU for “rule-out sepsis” and treated with empiric antibiotics for a minimum of 48 hours. The length of treatment was guided by laboratory tests, including complete blood count (CBC), C-reactive protein (CRP), and blood culture results. In the setting of maternal pretreatment with antibiotics for chorioamnionitis, the blood culture’s reliability has been questioned and, in many cases, obligated the newborn to an extended course of antibiotics.^1^ This historical practice, based on available evidence, including national guidelines for the prevention of Group B Streptococcal disease and empiricism borne from the lack of reliable tools to distinguish the infected from the noninfected infant, resulted in overtreatment with antibiotics.^2,3^ The intention was to prevent the dire consequences of morbidity and mortality from unrecognized neonatal sepsis. Our team universally recognized that the overwhelming majority of these infants did not have an infection. This fact and the evolving evidence of the harmful effects of antibiotic use in newborns, as well as the impact of delaying maternal–infant bonding, have made antibiotic stewardship for asymptomatic newborns a priority for our team.^4–11^ Although antibiotic use in newborns with infection is beneficial, routine antibiotic administration to newborns without infection exposes these infants to substantial risk with no demonstrable benefit to the individual infant.^12^

Our team targeted the management of EOS in asymptomatic term and near-term newborns as an opportunity to decrease the use of potentially unnecessary antibiotics. In January 2016, we engaged in a series of plan-do-study-act (PDSA) cycles to improve our antibiotic stewardship and safely decrease our use of empiric antibiotics in asymptomatic newborns older than 36 weeks. Our efforts relied heavily on evidence supporting the implementation of the neonatal EOS calculator.^13–15^ This report compares the impact of 3 successive PDSA ramps on antibiotic stewardship among asymptomatic newborns older than 36 weeks at risk of EOS at our center. Previous studies have demonstrated the incorporation of the neonatal EOS calculator into “rule-out sepsis” guidelines, specifically at academic medical centers.^16^ Our goal was to demonstrate the safety and success of implementing the EOS calculator in a smaller, community-based hospital.

## METHODS

Before January 2016, we admitted all term and near-term infants older than 36 weeks with sepsis risk factors, including maternal suspected chorioamnionitis, to the NICU. These infants had a CBC, CRP, and blood culture drawn on admission and were started on empiric antibiotic treatment with ampicillin and gentamicin. Repeated CBC and CRP were also obtained at 24 hours of life. If the infant remained asymptomatic with a negative blood culture, normal CBCs (defined as immature neutrophil to total neutrophil (I/T) ratio of *<* 0.2, WBC > 5,000 or ANC > 1,000) and normal CRPs (defined as CRP < 2), the antibiotics were discontinued at 48 hours.

**Fig. 1.**
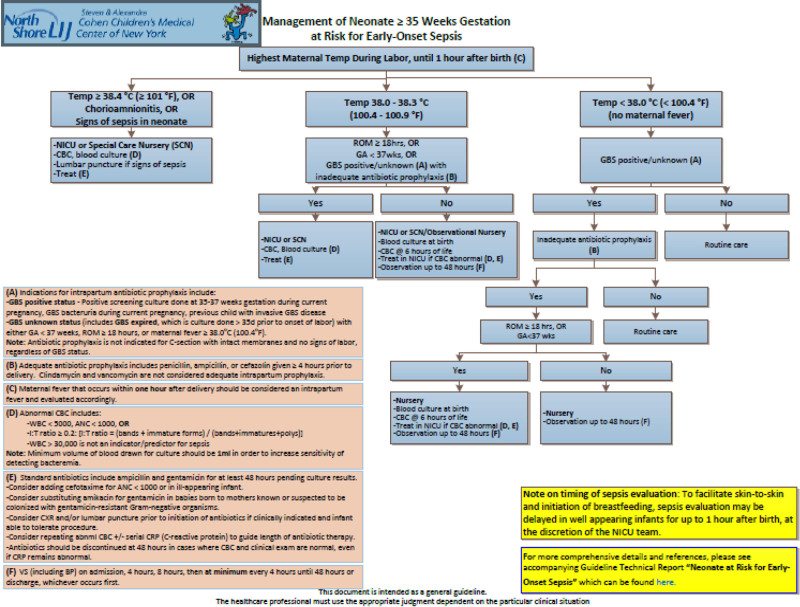
EOS Guideline based on 2012 AAP COFN Guidelines. Developed in collaboration with the Northwell Health Neonatal Service Line. Implemented January 2016 as part of PDSA ramp 1 at Lenox Hill Hospital.

In January 2016, we sought to improve our practice by instituting a newborn sepsis protocol based on the 2012 American Academy of Pediatrics (AAP) Committee on the Fetus and Newborn (COFN) guidelines (PDSA cycle 1) (Fig. 1). This initiative was an effort to standardize our protocol in collaboration with the Northwell Health Neonatal Service Line, of which we are a member hospital. Under this protocol, infants born to mothers with suspected chorioamnionitis were admitted to the NICU and managed according to maternal temperature. If the maternal temperature was 38.0–38.3 °C, a blood culture was obtained on admission and a CBC at 6 hours of age. Infants with a normal CBC were transferred to the well-baby nursery (WBN). Infants with an abnormal CBC were empirically treated with antibiotics pending blood culture results for a minimum of 48 hours. If the maternal temperature was *>*38.4 °C, a blood culture was obtained on admission, CBC at 6 hours, and empiric antibiotics were administered for at least 48 hours. Length of therapy was extended if the infant exhibited symptoms, the blood culture was positive, or the CBC was deemed suspicious for clinical sepsis. This intervention was our protocol during our first PDSA ramp (period 1: January 2016–June 2017).

**Fig. 2.**
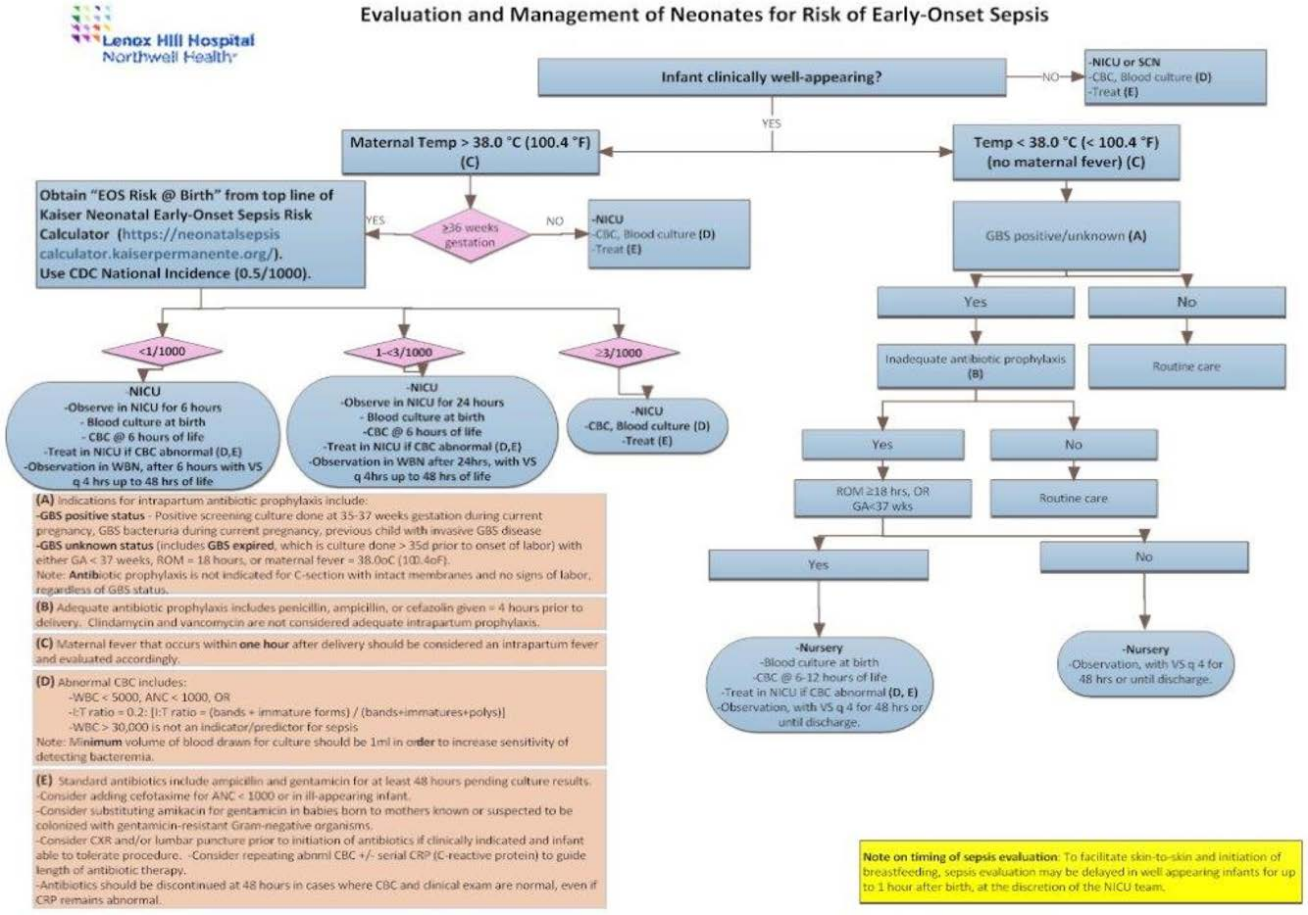
EOS Guideline integrating EOS calculator. Implemented in June 2017 as part of PDSA ramp 2 at LHH.

However, by December 2016, preliminary data demonstrated that although our practice had decreased NICU length of stay (LOS), we had not changed the antibiotic utilization rate among asymptomatic newborns older than 36 weeks gestation. In PDSA ramp 2, we revised our guidelines and integrated the neonatal EOS risk calculator score at birth in our algorithm (Fig. 2). The new algorithm was implemented in June 2017. Our goal was to decrease antibiotic use in our population of asymptomatic newborns older than 36 weeks by 25%. To ensure patient safety, we continued to admit at-risk neonates to the NICU. We judged our neonatal staff (nurses, nurse practitioners, physician assistants, and physicians) the most skilled to evaluate newborns at risk for sepsis and gauging illness and symptomatology. Management in the NICU was based on the EOS risk score. Neonatal staff observed infants with an EOS risk score of <1/1,000 in the NICU for a minimum of 6 hours. A blood culture was drawn on admission, and a CBC was sent at 6 hours of age. If the CBC was normal, the infant was transferred to the WBN and permitted to remain in the mother’s room to practice nonseparation. In the WBN, nursing staff and pediatric hospitalists closely observed at-risk infants with vital signs every 4 hours for 48 hours. No infants were discharged before 48 hours of life. Infants with an EOS risk score of 1–3/1,000 were observed in NICU for at least 24 hours. Blood culture was drawn on admission, and a CBC was sent at 6 hours of age. If the CBC was normal, the infant was transferred to the WBN and/or mother’s room at 24 hours, where they were closely observed with vital signs every 4 hours for an additional 24 hours. Once again, none of these infants were discharged before 48 hours of age. Any infant with an EOS risk score >3/1,000 or an abnormal CBC (defined as I/T ratio of *>* 0.2, WBC < 5,000, or ANC < 1,000) received empiric antibiotics for 48 hours pending blood culture results. This second PDSA ramp ran from June 2017 to February 2018 (period 2).

**Fig. 3.**
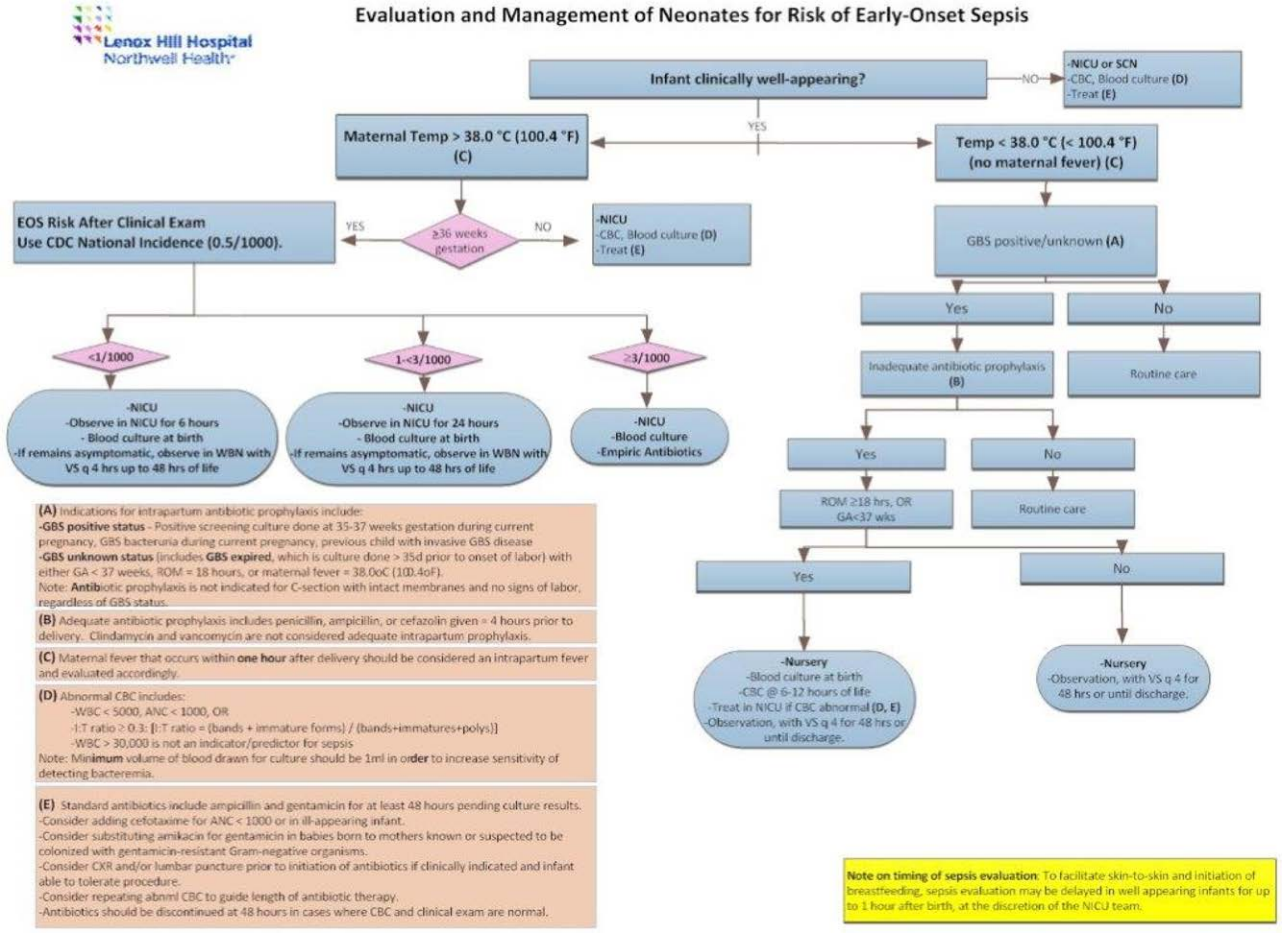
EOS Guideline integrating EOS calculator after clinical examination. This Guideline also omitted the routine use of CBCs in the evaluation of sepsis risk and the need for empiric Antibiotics. Implemented in February 2018 as part of PDSA ramp 3 at LHH.

Our third PDSA ramp began in February 2018. We further modified our existing algorithm with individualized newborn assessment by substituting the EOS risk after clinical examination score for the EOS risk at birth score. Once again, we sought to further decrease the empiric use of antibiotics in asymptomatic infants older than 36 weeks by an additional 25%. Our review of PDSA ramp 2 noted that a significant number of asymptomatic infants were empirically treated with antibiotics secondary to an abnormal CBC (ie, I/T ratio > 2). Subsequently, we omitted the routine use of CBC in determining the need for empiric antibiotics in PDSA ramp 3. Our third PDSA ramp was from February 2018 to December 2018 (period 3) (Fig. 3).

For the 4 periods, we retrospectively reviewed the number of asymptomatic term and near-term newborns older than 36 weeks born at Lenox Hill Hospital and those admitted to the NICU for the evaluation of EOS. Data collected included maternal fever, neonatal EOS symptoms, the number of infants with an abnormal CBC, the EOS score, the number of infants treated with antibiotics, and the number of infants with positive blood culture results, LOS in NICU, and exclusive breastfeeding. Our primary outcome measure was the percentage of asymptomatic newborns older than 36 weeks gestation treated with antibiotics during their evaluation for EOS. Secondary outcomes were the LOS in NICU and exclusive breastfeeding. Pairwise comparisons were accomplished using post hoc chi-square analysis. We monitored the monthly variation in asymptomatic 16 newborns older than 36 weeks who received antibiotics using statistical process control and adjusted centerlines based on the 8-points above or below the centerline rule.

As this was a quality initiative, Institutional Review Board approval was not necessary.

## RESULTS

### Baseline Data

In the year before period 1, 3,883 asymptomatic newborns older than 36 weeks were born at LHH, and 156 of these infants were admitted to the NICU solely based on sepsis risk factors. All 156 infants received antibiotics. This population represented 4.02% of asymptomatic newborns older than 36 weeks born at Lenox Hill Hospital during this period. There were no positive blood cultures.

### Period 1

During PDSA ramp 1, 5,041 asymptomatic newborns older than 36 weeks were born at LHH, and 275 of these infants were admitted to the NICU solely based on sepsis risk factors, predominately chorioamnionitis. During PSDA ramp 1, 219 infants were treated with antibiotics. These infants represented 4.34% of all asymptomatic newborns older than 36 weeks born at Lenox Hill Hospital during this period. Nine infants received antibiotics for >48 hours. There was one positive blood culture (*Streptococcus bovis*).

### Period 2

**Fig. 4.**
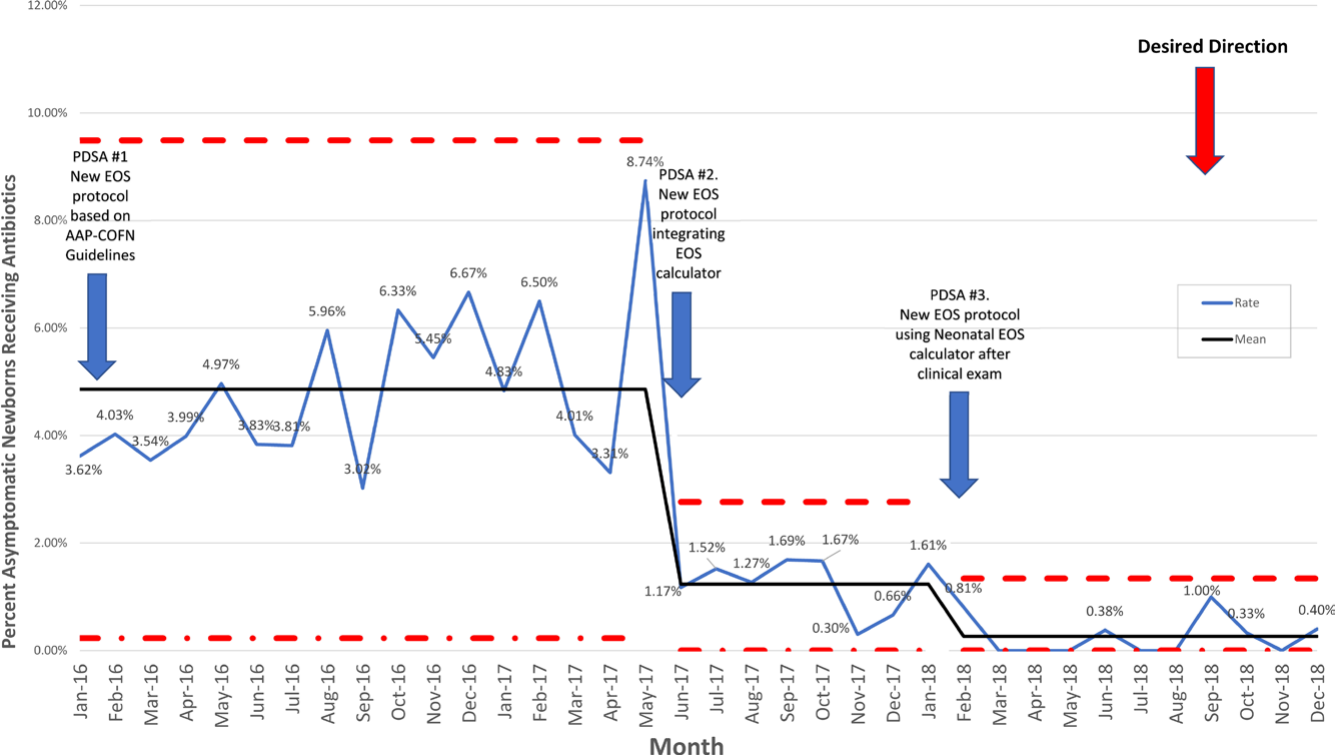
Control chart for PDSA ramps 1–3. Demonstrates percentage of asymptomatic newborns older than 36 weeks who received antibiotics from January 1, 2016–December 31, 2018.

During PDSA ramp 2, 2,581 asymptomatic newborns older than 36 weeks were born at LHH, and 109 of these infants were admitted to the NICU solely based on sepsis risk factors. Thirty infants were treated with antibiotics. This group represented 1.16% of all asymptomatic newborns older than 36 weeks born at Lenox Hill Hospital during this period. Our control chart demonstrates that this was a significant change in outcome (Fig. 4). During this period, one infant had a positive blood culture (*Escherichia coli*) and received >48 hours of antibiotics.

### Period 3

During PDSA ramp 3, 3,158 asymptomatic newborns older than 36 weeks were born at LHH, and 142 of these infants were admitted to the NICU solely based on sepsis risk factors. Three infants received antibiotics for 48 hours: One infant had an abnormal CBC, and 2 infants had positive blood cultures judged to be contaminants (*Bacillus* species and *Sphingomonas paucimobilus*). Additionally, one infant received antibiotics >48 hours secondary to EOS risk >3/1,000. These infants represented 0.13% of all asymptomatic newborns older than 36 weeks born at Lenox Hill Hospital during this period. Our control chart demonstrates that this was a significant change in outcome (Fig. 4).

### Primary Outcome

Comparing period 1 to period 2, the percentage of asymptomatic infants older than 36 weeks treated with antibiotics decreased by 73% (95% confidence interval 61%–82%; *P* < 0.0001). From period 2 to period 3, the percentage of asymptomatic infants older than 36 weeks treated with antibiotics decreased by an additional 89% (95% confidence interval 69%–96%; *P* < 0.0001). During the baseline period, the percentage of asymptomatic term and near-term infants older than 36 weeks treated with antibiotics was 4.02%; during period 1, it was 4.34%; during period 2 was 1.16%, and during period 3, it was 0.13% (*P* < 0.0001) (Table 1).

**Table 1.** Newborns Admitted to the LHH NICU Secondary to Maternal Fever among Asymptomatic Newborns Older Than 36 Weeks January 1, 2015, to December 31, 2018

Recommended Newborn Management	Baseline January 1, 2015, to December 31, 2015, N = 3,883	PDSA #1 January 1, 2016 to June 4, 2017, N = 5,041	PDSA #2 June 5, 2017 to February 11, 2018, N = 2,581	PDSA #3 February 12, 2018 to December 31, 2018, N = 3,158
Admission for 48 h blood culture, CBC, antibiotics × 48 hours	148	210	29	3
Admission for 7 d Blood culture, CBC Antibiotics × 7 d	8	9	1	1
Vital signs q 4 and 6 h in NICU 42 h in WBN blood culture, Cbc No antibiotics	N/A	N/A	64	129*
Vital signs q 4 and 24 h in NICU 24 h in WBN blood culture, CBC no antibiotics	N/A	N/A	45	13*
Admission for 6 h blood CULTURE No antibiotics CBC at 6 h of life	N/A	56	N/A	N/A
Total No. infants treated with antibiotics	156 (4%)	219 (4.3%)†	30 (1.16%)†	4 (0.12)†

*No CBC

†*P* < 0.0001.

### Secondary Outcomes

Mean LOS in the NICU for asymptomatic newborns admitted for sepsis evaluation was 47.2 hours during period 1, 26.5 hours during period 2, and 15.6 hours for period 3. Among these same infants, exclusive breastfeeding at discharge was 29% in period 1, 39% during period 2, and 44% during period 3.

### Balancing Metric

During period 1, there were no missed cases of infection and no delays in antibiotics. During period 2, there were no missed cases of infection, but there was a delay in treatment of 12 hours of one infant with a positive culture for *E. Coli*. This infant was asymptomatic during his entire hospital stay, and the positive culture likely represented transient bacteremia. During period 3, there were no missed cases of infection and no delays in treatment. During periods 1–3, no newborns were readmitted to our hospital for missed EOS.

## DISCUSSION

Empirically treating all asymptomatic infants at risk for EOS based solely on risk factors grossly overexposed the newborn population to unnecessary antibiotics. The integration of the EOS risk score at birth to our AAP COFN-based guidelines allowed our team to reduce antibiotic use among asymptomatic infants older than 36 weeks by 73%. Adopting the EOS risk score after clinical examination and omitting the routine use of CBCs in evaluating asymptomatic infants at risk for EOS further reduced the number of asymptomatic infants treated by an additional 89%. This change in our guidelines for treating EOS in asymptomatic infants older than 36 weeks enabled our team to defer the use of antibiotics in almost all asymptomatic infants safely. By safely deferring antibiotic use, we speculate that we have prevented both potential short- and long-term antibiotic-associated health impacts while improving the maternal-newborn hospitalization’s short-term outcomes. In particular, we speculate that we decreased painful procedures for the newborn, complications such as intravenous infiltrates, and minimized aminoglycoside exposure impacts.

Importantly, we submit that we decreased the infant’s separation from mother and other family members by using these guidelines. We should have a measurable impact on successful breastfeeding, maternal–infant bonding, and patient/family satisfaction with the birth experience. In our cohort, we found that among our asymptomatic newborns older than 36 weeks admitted to the NICU for sepsis evaluation, NICU LOS decreased by 67%, and exclusive breastfeeding increased by 50% throughout our 3 PDSA cycles.

Future PDSA cycles will focus on improving nonseparation by transitioning the observation of infants with sepsis risk of <1/1,000 to the newborn nurseries and instituting a more selective practice of obtaining blood cultures.

## CONCLUSIONS

As a result of multiple PDSA ramps at our center, we were able to implement sepsis guidelines which have reduced the use of antibiotics among term and near-term asymptomatic newborns older than 36 weeks by 97%. We must emphasize, however, that our guidelines require initial evaluation of all infants at risk in the NICU for a minimum of 6 hours, admission blood cultures from every infant, as well as serial examination for the first 48 hours of life. These interventions may not be practical or acceptable at other centers. Nevertheless, by using individualized assessment guidelines based on the integration of the 2012 AAP COFN and the EOS calculator, in our center, we have essentially eliminated the use of antibiotics among term and near-term asymptomatic newborns older than 36 weeks at risk for EOS without evidence of missed EOS or adverse events. Our center’s experience demonstrates that Neonatal EOS Guidelines can be changed to incorporate the EOS calculator and significantly decrease antibiotic utilization in asymptomatic, otherwise healthy newborns older than 36 weeks. Although this practice may be standard of care in many larger academic institutions, many smaller community hospitals may be hesitant to defer antibiotics. We share our example of safe implementation with significant benefits for our newborns and their families.

## DISCLOSURE

The authors have no financial interest to declare in relation to the content of this article.
